# Ten simple rules for the sharing of bacterial genotype—Phenotype data on antimicrobial resistance

**DOI:** 10.1371/journal.pcbi.1011129

**Published:** 2023-06-22

**Authors:** Leonid Chindelevitch, Maarten van Dongen, Heather Graz, Antonio Pedrotta, Anita Suresh, Swapna Uplekar, Elita Jauneikaite, Nicole Wheeler

**Affiliations:** 1 MRC Centre for Global Infectious Disease Analysis, Imperial College, London, England, United Kingdom; 2 AMR Insights, Amsterdam, the Netherlands; 3 Biophys Ltd, Usk, Wales, United Kingdom; 4 FIND, the global alliance for diagnostics, Geneva, Switzerland; 5 NIHR HPRU in Healthcare Associated Infections and Antimicrobial Resistance, Imperial College, London, England, United Kingdom; 6 Institute of Microbiology and Infection, University of Birmingham, Birmingham, England, United Kingdom; Dassault Systemes BIOVIA, UNITED STATES

## Abstract

The increasing availability of high-throughput sequencing (frequently termed next-generation sequencing (NGS)) data has created opportunities to gain deeper insights into the mechanisms of a number of diseases and is already impacting many areas of medicine and public health. The area of infectious diseases stands somewhat apart from other human diseases insofar as the relevant genomic data comes from the microbes rather than their human hosts. A particular concern about the threat of antimicrobial resistance (AMR) has driven the collection and reporting of large-scale datasets containing information from microbial genomes together with antimicrobial susceptibility test (AST) results. Unfortunately, the lack of clear standards or guiding principles for the reporting of such data is hampering the field’s advancement. We therefore present our recommendations for the publication and sharing of genotype and phenotype data on AMR, in the form of 10 simple rules. The adoption of these recommendations will enhance AMR data interoperability and help enable its large-scale analyses using computational biology tools, including mathematical modelling and machine learning. We hope that these rules can shed light on often overlooked but nonetheless very necessary aspects of AMR data sharing and enhance the field’s ability to address the problems of understanding AMR mechanisms, tracking their emergence and spread in populations, and predicting microbial susceptibility to antimicrobials for diagnostic purposes.

## Author summary

The growing worldwide threat of antimicrobial resistance (AMR) makes the sharing of resistance data a priority for both researchers and public health practitioners. In particular, the growth of high-throughput sequencing data, in conjunction with AMR phenotypes, has the potential to revolutionise AMR diagnostics and surveillance. However, there is a significant heterogeneity in the ways that this type of data is currently shared, which makes it challenging to perform its analysis at scale. As both producers and users of publicly available genotype–phenotype data on AMR, we propose 10 simple rules that can mitigate this situation and nudge the field towards the adoption of better practices.

## Introduction

Antimicrobial resistance (AMR), the phenomenon whereby microbes successfully evade the action of antimicrobial drugs designed to kill them or stop their growth, is a growing public health threat worldwide, with recent estimates of over 1.27 million deaths directly attributable to it [1].

The emergence of high-throughput sequencing, used in conjunction with computational methods such as bioinformatics, mathematical or statistical modelling, and machine learning, holds the promise of improving surveillance, enabling more accurate diagnosis, and informing treatment decisions within the infectious disease realm [2].

However, the successful use of next-generation sequencing (NGS) and accompanying computational methods, especially those based on machine learning, for the control of AMR is critically reliant on the availability of large-scale, high-quality data that details both genotypes (data on the microbe’s genome) as well as phenotypes (antimicrobial susceptibility tests, or AST) for individual isolates.

Unfortunately, although microbial data are widely considered as falling outside patient privacy considerations [3], barriers remain for collecting, sharing, and using this type of data, which result from the variety of options and standards for publishing genotypic and phenotypic data.

We propose 10 simple rules for the effective collecting and sharing of genotype–phenotype AMR data within a publication so as to make it easy-to-use for downstream studies involving computational analysis and the robust identification of genomic determinants of AMR. We recognise that sharing this type of data is not always a primary motivation for collecting it; however, the benefits of sharing data for both the research group and the field as a whole are well documented [4,5], while the additional effort required can be limited to a small upfront investment. We emphasise that, while following these recommendations will make downstream analysis and data integration easier, the most important contribution from a group collecting such data is to make it fully available via open-access journals, databases, or repositories.

### Rule 1: Decide on a well-defined format; provide all data in this format

The lack of data standardisation is a key barrier to useful inference that can inform AMR control [6]. A number of standard formats (such as those by GMI [7], NCBI [8], and WHONET [9]) and guidelines (such as those by the GSC [10] and PHA4GE [11]) already exist for publishing genotype and phenotype data, as well as metadata (relevant information about the isolate, such as the date, location of isolation, and the source of the sample). While the community has yet to reach agreement on which of these formats should be adopted, each group or research consortium should internally agree on a specific format so that, at a minimum, all the data from the group can be easily compared, combined, and analysed together.

Each of the fields in Box 1 above should appear in its own column, with some fields potentially subdivided into several columns; for example, genus and species. Further, whenever abbreviations or other ambiguous notation is used, a separate data dictionary should be provided to enable a disambiguation to take place in downstream analysis. Note that the last bullet point means that the common approach consisting of reporting either “Sensitive”or listing the drugs to which the isolate is resistant should be avoided.

**Box 1. Recommended format for reporting genotype and AMR phenotype data**

Based on a review of the available formats, we recommend the use of a single file in a tabular format, with 1 row per isolate, and the following information made available for each one:

- Internal ID (this can be helpful as the key for merging genotype and phenotype tables)
- Accession number for the raw genotypic data in databases (NCBI, ENA, and DDBJ [12])
- Additional accession numbers specific to the isolate, such as the assembled contigs
- Collection date, in a “long format” (e.g., 12 October 2022) to avoid potential confusion
- Collection location, ideally in an unambiguous format such as longitude and latitude
- Source of isolation (animal, clinical, environmental, etc.)
- For clinical isolates, the fluid or tissue the isolate is from (blood, sputum, stool, urine, etc.)
- Isolate genus and species
- Experimental approach used to measure phenotypic susceptibility (agar dilution, Etest, Vitek2, etc. [13])
- For each drug or combination tested for susceptibility, ideally the 3 columns specified in Rule 4, otherwise 1 column with the resistance status (susceptible (S), intermediate susceptibility (I), resistant (R))

While the adoption of a uniform standard may seem onerous, our experience suggests that this pays long-term dividends in the form of easier sharing, less potential for confusion in downstream analysis, and a greater incentive for other groups to use the data, which generates additional credit in exchange of only minimal additional effort.

### Rule 2: Provide relevant contextual sample metadata

**Fig 1:**
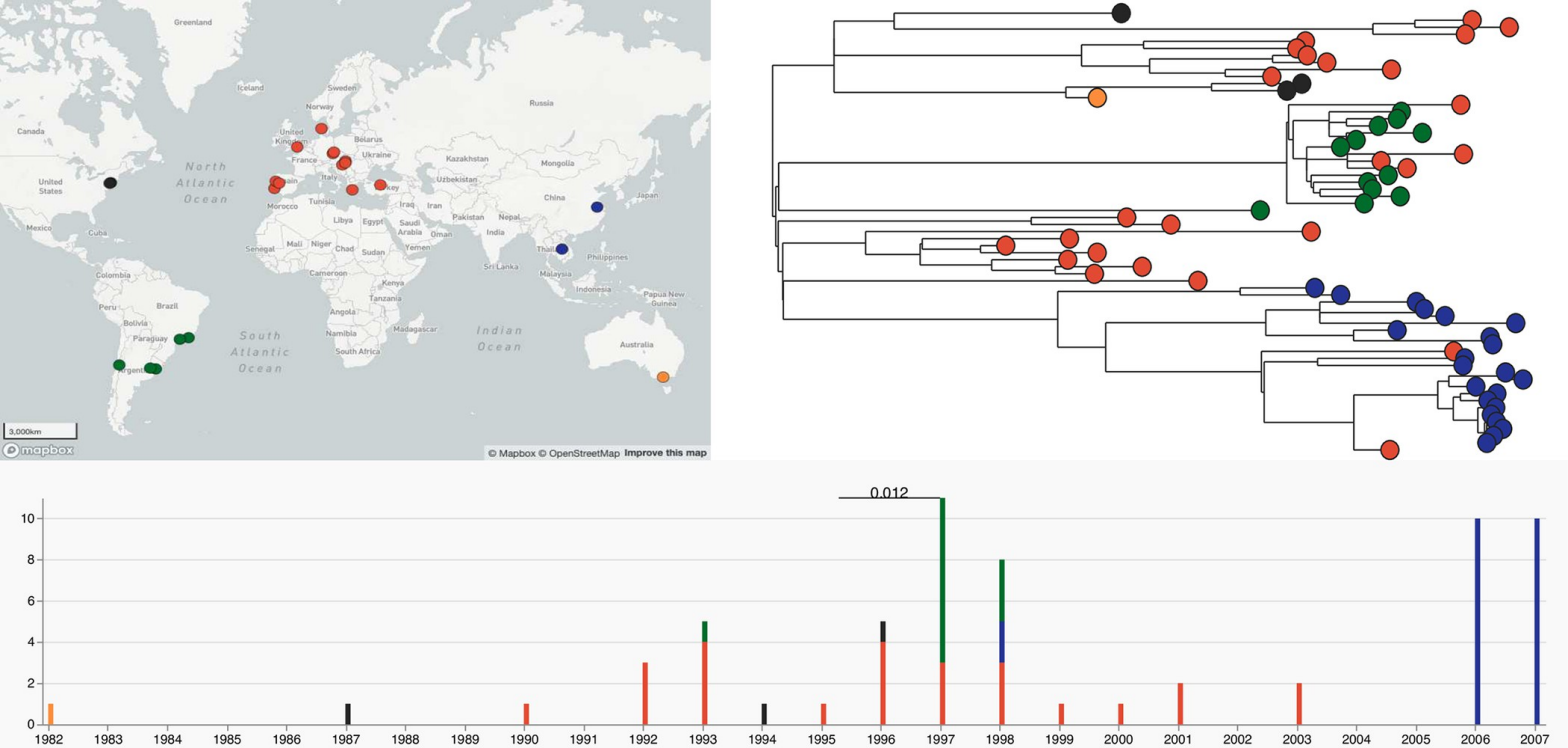
Microreact visualisation. An example of the informative use of location and sample collection date for data contextualisation. Left: Map. Right: Phylogenetic tree. Bottom: Timeline. Microreact showcase, Global *Staphylococcus aureus* ST239 [14,15].

Include available relevant clinical information on the samples, such as whether bacterial samples were taken from blood, urine, the environment, etc, as for certain microbes the interpretation of MICs or drugs tested will depend on the source of the isolate. Date and location of collection are also important, with latitude and longitude being pivotal for visualising and contextualising the location of different AMR determinants, patterns, and high-risk strains, as shown on a small example dataset in Fig 1 below.

### Rule 3: Make all samples identifiable, including those from externally sourced studies

When documenting genotypic and phenotypic data on isolates from other studies, provide individual accession numbers for each sample in the metadata table, alongside an overall reference for each study included. This allows more streamlined retrieval of data and prevents mismatching of samples due to incompatible IDs used in publication metadata and online sequence data repositories, as well as correctly attributes credit to the data producers.

### Rule 4: Provide raw quantitative data for phenotypic AST results

A number of factors can impact the quality and trustworthiness of AST [16], such as the depth and composition of media, spacing and potency of antibiotic sources, and incubation time and temperature. Internal and external quality control (QC) of AST should ideally be performed [16] to ensure that phenotyping results are consistent with those obtained in gold-standard laboratories.

The correct interpretation of a minimum inhibitory concentration (MIC) involves factors such as the pharmacokinetics of an antimicrobial and the body site of an infection. As a result, the categorisation [17] of quantitative MIC data as susceptible (S), intermediate (I), or resistant (R) can depend on the infection context. Furthermore, intermediate results may correspond to both a combination of strains that can be successfully treated with an increased dosage of the antibiotic as well as cases falling into a well-known buffer zone [17] (also known as an area of technical uncertainty, or ATU [18]) in which results from a test should be interpreted with caution. Breakpoints for categorising isolates also change across continents (e.g., EUCAST [19] versus CLSI [20]) as well as time, meaning that categorical assignments may not be comparable between Europe and North America, or across different years. To ensure published data can be integrated with other sources, researchers should specify the breakpoints used to categorise isolates as S, I, or R, and provide the raw quantitative measurements (such as minimum inhibitory concentration, disk diffusion zone, or zone of inhibition size) in the following 3 columns:

- Measurement value
- Measurement sign (= for exact, < if below the minimum, or > if above the maximum)
- Measurement unit (typically, mg/L for concentrations or mm for diameters)

Different studies may also test a different range of MIC values, making comparison of results challenging. To account for these reporting differences, maximum and minimum MICs tested for each antibiotic should be reported explicitly, either as an additional column or in a separate table. This would allow other researchers to appropriately preprocess their results and compare accuracy within the range covered by both studies. Lastly, the community working on AMR would greatly benefit from the genotype–phenotype data being reported for a standardised panel of antibiotics specified for the bacterial pathogen being studied. As many studies are opportunistic and rely on clinically generated MIC data, this may not always be possible. However, only testing all the isolates of a particular pathogen in a study against the same panel of antibiotics can guarantee to provide a complete, internally comparable dataset.

### Rule 5: Include the phenotyping method

The phenotyping method used should be included as a metadata column in the same data source as the phenotypic data. Each phenotyping method has differing strengths and weaknesses [17] in terms of flexibility in antibiotics and concentrations tested, error rates, scalability, and affordability. Methods may differ in accuracy for specific microbe–drug combinations and taking this into account is important for comparative studies and evaluating discrepancies between genotype and phenotype. For example, Vitek2 is associated with higher error rates when measuring cefepime resistance in ESBL-producing *Escherichia coli* [21], and a range of phenotyping methods have been found to have high error rates compared to agar dilution when measuring fosfomycin resistance in ESBL-producing *E*. *coli* [22]. Knowing exactly which method has been used as an additional column in the supplementary material or metadata is helpful, especially when samples are processed with different methods.

### Rule 6: Share tabular data files in machine-readable format

It is best to submit supplementary files with raw data and metadata in a format that can be easily read and combined with other data. Of all such formats, the 2 most easily accessible ones are tab-separated values (.tsv) and comma-separated values (.csv), with multiple open-source tools supporting their use without any licensing requirements. The choice between.tsv and.csv is largely a matter of preference. The.csv format requires careful handling when a comma is also used as a separator within a field (e.g., longitude, latitude in the location data), as well as in some locales and operating systems that use semicolons rather than commas as separators. On the other hand, the.csv format is currently accepted by a larger number of journals than the.tsv format.

Microsoft Excel formats (.xls or.xlsx), while convenient, create several additional challenges caused by automated conversions. Sample IDs such as 8E12 may be interpreted as numbers in scientific notation; some gene names may be interpreted as dates (although this problem is largely limited to human genes) [23], and bacterial datasets may exceed the size limit in Excel [24].

Image formats such as.png or.jpg and the portable data format (.pdf) are the least appropriate for sharing data. While phylogenies annotated with isolate IDs and AST results at the tips are common in publications describing AMR genotype and phenotype data, they are challenging to extract into a machine-readable format despite the advances in optical character recognition (OCR) technology [25]. If such a phylogeny is included as an image, the tip data and metadata should also be included as a tabular file, or the phylogeny itself provided in a machine-readable format (e.g., Newick [26]).

The file(s) containing the data in tabular format can either be included as supplementary files or, especially for large files, deposited in an openly accessible repository that provides a permanent digital object identifier (DOI), such as figshare, Synapse, or Zenodo. Alternatively, a project can be created on the Microreact platform [27] (also see Fig 1), enabling both an easy visualisation of the data’s accompanying metadata as well as access to the underlying data in tabular format.

### Rule 7: Make raw genomic data available

Submit raw reads, not just assemblies, whenever possible, and when submitting data to common databases such as NCBI, ENA, or DDBJ [12], provide experiment numbers, not only accession numbers, in the data table. Specify the machine and software used for base calling, including settings, and specify what, if any, QC steps were taken to process the raw data. When reporting data on isolates included in the study, include isolates that were sequenced and subsequently excluded by QC, with a column distinguishing isolates that were included and those that were not. An ongoing effort by the Data Structures Working Group within the Public Health Alliance for Genomic Epidemiology (PHA4GE) has resulted in the creation of a flexible yet precise ontology of flags that can be used to identify QC issues [28].

The inclusion of all genotypic data allows recovery of excluded isolates in later studies that may use different QC criteria, as well as improved calibration of analytical tools for sequencing data. As some sequencing platforms such as Oxford Nanopore (ONT) refine their chemistries and base calling algorithms, providing traces and chemistry information can also allow better assessment of the data and creates the potential for subsequent correction of biases introduced through different base calling methodologies, thus facilitating the inclusion of older data in an analysis.

### Rule 8: Make genotypic resistance calls in a reproducible manner

When reporting the presence of resistance determinants, these genotypic calls should be easily reproducible by using the same workflow. A popular option for annotating resistance genotypes is via an open database of resistance determinants, e.g., CARD [29] for bacteria or MARDy [30] for fungi—see Table 1 in Kaprou and colleagues [31]. As some of these are regularly updated, the name and version of the database must be specified. Furthermore, AMR can be facilitated by the expression of an acquired AMR gene or by a chromosomal mutation in an antibiotic target [32]. It is thus important to specify the type of AMR determinant involved when possible.

Effort should also be made to use established nomenclature to describe the resistance gene, such as the NCBI’s formalised process for naming beta-lactamase genes [33] or CARD’s Antibiotic Resistance Ontology and Short Names conventions [29]; this will ensure that the report is easily findable in relevant searches. The hAMRonization tool [34], published by PHA4GE, can also assist the data producers in annotating the identified genes in a systematic way. If genotype calls were made using a custom genomic determinant database, the database and the analysis code should also be supplied in a format that can easily be used by a reader. When AMR genes have been detected by PCR rather than NGS, this should be made clear in the data table, and the primers used should be provided explicitly, as PCR may miss variants of a gene or incorrectly conflate variants of a gene [35].

### Rule 9: Report novel resistance determinants in a systematic way

The identification of new resistance determinants such as SNPs, indels, copy numbers, or alleles of specific genes [29] can be a valuable tool for tracking the de novo emergence and spread of resistance mechanisms. But these mechanisms are only detectable in other datasets if, in addition to the variant being described in the main text of a paper, the full relevant sequence information is given in the supplementary materials. Ideally, the determinant’s complete genomic sequence should also be deposited in an open-access database of genetic determinants of AMR and the accession number provided. For example, to meet the current criteria for inclusion into CARD, an AMR determinant must be described in a peer-reviewed scientific publication, its DNA sequence available in GenBank, and there must be clear experimental evidence of elevated MIC over controls [29].

### Rule 10: Share the data to the fullest extent possible

While all of the above rules should be followed in an ideal scenario, we recognise that time, cost, and expertise constraints, as well as the time-sensitive nature of data from ongoing or emerging outbreaks, or of metadata that contains protected clinical or public health information, may create substantial practical barriers to their implementation. For this reason, we recommend that researchers share their genotype and phenotype AMR data and metadata to the full extent they can do so without adverse consequences, in the format and database of their choice, because the only thing worse than having data in an unusual format or with inconsistent annotations is not having any data at all.

Thankfully, a variety of initiatives are underway to facilitate AMR data sharing, from software solutions such as WHONET [9] and Microreact [27], to community efforts such as the Pathogenwatch resource for *Neisseria gonorrhoeae* [36]. The experience of the GISAID platform during the COVID-19 pandemic, with over 5 million genotypes deposited with accompanying metadata in less than 2 years [37], suggests that scalable pathogen genomic data sharing is possible, and it is our hope that the field of AMR will take inspiration from this building momentum.
